# Serum Free Fatty Acids Independently Predict Adverse Outcomes in Acute Heart Failure Patients

**DOI:** 10.3389/fcvm.2021.761537

**Published:** 2021-12-22

**Authors:** Yi Yu, Chunna Jin, Chengchen Zhao, Shiyu Zhu, Simin Meng, Hong Ma, Jian'an Wang, Meixiang Xiang

**Affiliations:** Department of Cardiology, The Second Affiliated Hospital, Zhejiang University School of Medicine, Hangzhou, China

**Keywords:** acute heart failure, free fatty acids, Lipolysis, Mortality, rehospitalization

## Abstract

**Background:** Perturbation of energy metabolism exacerbates cardiac dysfunction, serving as a potential therapeutic target in congestive heart failure. Although circulating free fatty acids (FFAs) are linked to insulin resistance and risk of coronary heart disease, it still remains unclear whether circulating FFAs are associated with the prognosis of patients with acute heart failure (AHF).

**Methods:** This single-center, observational cohort study enrolled 183 AHF patients (*de novo* heart failure or decompensated chronic heart failure) in the Second Affiliated Hospital, Zhejiang University School of Medicine. All-cause mortality and heart failure (HF) rehospitalization within 1 year after discharge were investigated. Serum FFAs were modeled as quartiles as well as a continuous variable (per SD of FFAs). The restricted cubic splines and cox proportional hazards models were applied to evaluate the association between the serum FFAs level and all-cause mortality or HF rehospitalization.

**Results:** During a 1-year follow-up, a total of 71 (38.8%) patients had all-cause mortality or HF rehospitalization. The levels of serum FFAs positively contributed to the risk of death or HF rehospitalization, which was not associated with the status of insulin resistance. When modeled with restricted cubic splines, the serum FFAs increased linearly for the incidence of death or HF rehospitalization. In a multivariable analysis adjusting for sex, age, body-mass index, coronary artery disease, diabetes mellitus, hypertension, left ventricular ejection fraction and N-terminal pro-brain natriuretic peptid, each SD (303.07 μmol/L) higher FFAs were associated with 26% higher risk of death or HF rehospitalization (95% confidence interval, 2–55%). Each increasing quartile of FFAs was associated with differentially elevated hazard ratios for death or HF rehospitalization of 1 (reference), 1.71 (95% confidence interval, [0.81, 3.62]), 1.41 (95% confidence interval, [0.64, 3.09]), and 3.18 (95% confidence interval, [1.53, 6.63]), respectively.

**Conclusion:** Serum FFA levels at admission among patients with AHF were associated with an increased risk of adverse outcomes. Additional studies are needed to determine the causal-effect relationship between FFAs and acute cardiac dysfunction and whether FFAs could be a potential target for AHF management.

## Introduction

Heart failure (HF) has become a major and growing public health challenge with a worldwide prevalence of 64.3 million cases (1). Over the last 30 years, despite tremendous advances in the management of chronic HF, the central treatment of AHF remains symptomatic but less satisfactory due to the high heterogeneity of underlying etiology, resulting in a remarkably high risk of mortality and rehospitalization (2, 3).

The normal heart function predominantly relies on fatty acid (FA) oxidation, responsible for 60–90% of ATP production (4). Since the *de novo* synthesis of FAs is inactive in the heart, FA oxidation largely depends on the uptake of circulating free fatty acids (FFAs), the byproducts of lipolysis (5). Although FFAs serve as the major energy substrate for the heart, high circulating FFA levels may exert deleterious effects on the heart. FA metabolism requires more oxygen than other substrates like glucose or ketone bodies, which potentially exacerbates the hypoxia injury under some pathological conditions (6). Moreover, elevated FFAs lead to the augmented myocardial FFAs uptake as well as excessive FA storage in cardiomyocytes, which drives cardiac lipotoxicity and causes myocardial damage (4). Thus, circulating FFAs may serve as a risk factor, hallmark, or potential target for diverse conditions.

Indeed, previous studies have shown that FFAs serve as a risk factor for obesity (7), diabetes (8), and coronary heart diseases (9, 10). Elevated FFAs are independently associated with compensatory HF (11, 12) and linked to higher 3-month mortality (13). A surge in catecholamines, inflammatory cytokines such as tumor necrosis factor (TNF)-alpha, and natriuretic peptides may, in part, explain the increased level of FFAs (9), which are all lipolytic inducers (14). In return, elevated FFAs can induce insulin resistance in cardiomyocytes and impair cardiac function as revealed by animal studies (15).

Considering FFAs are closely tied to fatty acid oxidation under exercise and stress (16), it is reasonable to propose that serum FFAs levels may provide important prognostic information for patients with AHF. It would be valuable for risk stratification and tailoring of therapy to optimize the management of AHF. However, more direct evidence for the prognostic value of FFAs in AHF is still lacking. We aim to determine whether higher levels of FFAs predict adverse outcomes among Chinese patients admitted with AHF.

## Methods

### Study Population

This is a single-center, observational cohort study. Patients admitted for AHF in Department of Cardiology, the Second Affiliated Hospital, Zhejiang University School of Medicine between January 2019 and December 2019 were enrolled. Ethics Committee approvals were obtained from the Institutional Review Board for Human Studies of Second Affiliated Hospital of Zhejiang University School of Medicine (<city>Hangzhou </city>, China), and verbal informed consent was obtained from the patients during telephone follow-up.

The diagnosis of AHF was based on the ESC Guidelines and required elevated N-terminal pro-B-type natriuretic peptide (NT-proBNP) concentrations ≥1,000 ng/L and only those whose duration from onset of AHF or symptom exacerbation ≤1 month were recruited. Those with severe renal failure (eGFR <30 mL/min·1.73 m^2^), renal replacement therapy, severe liver dysfunction (serum aminotransferase concentration more than 10 times above upper limit of normal range), serious infection, severe pulmonary diseases, systemic autoimmune disorder, malignancies, valvular disease, pregnancy, missing FFAs measurement or younger than 18 years were excluded in the study. Only those who survived to hospital discharge with complete full 1-year follow-up data (*n* = 183) were included in the analysis (Supplementary Figure 1).

### Definition of Events

The study endpoint was the composite of all-cause death and HF rehospitalization through the 1-year follow-up after discharge. The endpoint events were ascertained based on rehospitalization records and telephone contacts.

### Data Collection

Clinical data were extracted from electronic medical records. The laboratory parameters were measured at admission. For patients who received a laboratory test multiple times during this time period, only the first test results were included. Serum FFAs, low-density lipoprotein cholesterol (LDL-C), high-density lipoprotein cholesterol (HDL-C), total cholesterol (TC), triglycerides (TG), fasting glucose (FBG) and glycated hemoglobin A1c (HbA1c) were measured in an overnight fasting state, generally within 24 h after admission, while fasting status was unknown for the evaluation of the other laboratory parameters including N-terminal pro-brain natriuretic peptide (NT-proBNP), alanine aminotransferase (ALT), aspartate aminotransferase (AST), urea nitrogen (BUN) and creatinine. The measurements of serum FFAs, LDL-C, HDL-C, TG, TC, FBG, ALT, AST, BUN, and creatinine were performed on a Beckman Coulter instrument AU5800 (Beckman Coulter, Brea, California, USA). Serum levels of FFAs were analyzed using an enzymatic reagent (ACS-ACOD method) from LEADMAN (Beijing, China). The level of HbA1c was determined by a TOSOH HLC-723G8 automatic glycohemoglobin analyzer (Tosoh Corporation, Yamaguchi 746-0042, Japan). NT-proBNP was measured by electrochemiluminescence on an Cobas e801 (Roche Diagnostics, Rotkreuz, Switzerland). Echocardiography was used to measure left ventricular ejection fraction (LVEF), which was obtained during the hospital stay. Estimated glomerular filtration rate (eGFR) was estimated by the Chronic Kidney Disease Epidemiology Collaboration (CKD-EPI) formula. The triglyceride-glucose (TyG) index was calculated as the ln[fasting glucose level (mg/dL) × triglyceride level (mg/dL)/2].

### Statistical Analysis

Baseline characteristics were compared among quartiles of FFAs using ANOVA or Kruskal-Wallis test for continuous variables, depending on the data distribution, and χ2 for categorical variables. Continuous variables were presented as mean ± standard deviation (SD) or median [inter-quartile range (IQR)] if skewed. Categorical variables were presented as N (%). Correlation between two variables was examined by the Pearson or Spearman analysis. Cubic splines were utilized to evaluate the linearity of the relationship between FFAs and the incidence of study endpoints. Cox proportional hazards regression models were used to analyze the association between FFAs levels and time to event (only the first endpoint event was accounted in our analysis). FFAs were modeled as quartiles as well as a continuous variable (per SD increase of FFAs). Subgroup analysis of patients and outcomes and the receiver-operating curve (ROC) analysis were performed (presented in Supplementary Material). A two-tailed *P* < 0.05 was considered statistically significant. Statistical analyses were performed with SPSS statistical software (version 23.0) and R statistical software (version 4.1.0).

## Results

### Descriptive Analysis

A total of 183 patients with AHF were included in the study, with a median age of the study participants was 73 years (IQR: 63~79). The baseline demography was compared according to FFAs quartiles as shown in Table 1. After 1 year follow-up, 71 (38.8%) participants suffered from all cause of death (*n* = 13), or HF rehospitalization (*n* = 61). Participants who developed adverse events were with higher FFAs levels, consistent with higher NT-proBNP although the difference was not statistically significant (Supplementary Table 1). Interestingly, the total serum lipids were not significantly different among participants with different quartiles of FFAs.

**Table 1:** Characteristics of patients with acute heart failure at baseline by quartiles of serum free fatty acids.

	**All patients** ***n* = 183**	**Q1 (≤385.60)** ***n* = 46**	**Q2 (385.60–596.20)** ***n* = 46**	**Q3 (596.20–797.30)** ***n* = 46**	**Q4 (>797.30)** ***n* = 45**	***P*-value**
Age, years	73 (63, 79)	72 (65, 78)	76 (63, 82)	77 (69, 83)	66 (57, 73)	**<0.001**
Male sex, *n* (%)	117 (63.9)	28 (60.9)	31 (67.4)	30 (65.2)	28 (62.2)	0.915
Body-mass index, kg/m^2^	23.8 (20.8, 26.0)	22.2 (20.2, 25.4)	24.1 (22.0,26.3)	24.2 (20.8, 26.9)	24.2 (20.8, 25.3)	0.224
MAP, mmHg	90.7 (82.0, 100.7)	91.0 (82.8, 99.3)	89.3 (81.7, 94.8)	91.0 (79.0, 106.4)	91.7 (85.7, 104.0)	0.783
LVEF, %	35.3 (28.2, 48.5)	39.0 (30.2, 54.7)	34.3 (27.6, 55.3)	37.3 (28.5, 45.1)	32.0 (26.5,43.4)	0.197
NYHA functional class						0.078
II, *n* (%)	33 (18.0)	11 (23.9)	10 (21.7)	6 (13.0)	6 (13.3)	
III, *n* (%)	81 (44.3)	25 (54.3)	18 (39.1)	23 (50.0)	15 (33.3)	
IV, *n* (%)	69 (37.7)	10 (21.7)	18 (39.1)	17 (37.0)	24 (53.3)	
*De novo* HF, *n* (%)	47 (25.7)	13 (28.3)	12 (26.1)	10 (21.7)	12 (26.7)	0.905
Co-morbidities, *n* (%)						
Coronary artery disease	71 (38.8)	21 (45.7)	14 (30.4)	21 (45.7)	15 (33.3)	0.291
Diabetes mellitus	51 (27.9)	9 (19.6)	16 (34.8)	11 (23.9)	15 (33.3)	0.296
Hypertension	98 (53.6)	27 (58.7)	26 (56.5)	25 (54.3)	20 (44.4)	0.539
Atrial fibrillation/atrial flutter	87 (47.5)	18 (39.1)	21 (45.7)	25 (54.3)	23 (51.1)	0.483
Smoking	67 (36.6)	15 (32.6)	22 (47.8)	17 (37.0)	13 (28.9)	0.265
Medications during hospitalization, *n* (%)						
Intravenous Diuretics	128 (69.9)	26 (56.5)	33 (71.7)	32 (69.6)	37 (82.2)	0.065
Intravenous vasodilator	15 (8.2)	4 (8.7)	3 (6.5)	6 (13.0)	2 (4.4)	0.482
Intravenous vasopressor	6 (3.3)	0 (0)	1 (2.2)	2 (4.3)	3 (6.7)	0.317
Intravenous inotropic agent	54 (29.5)	12 (26.1)	14 (30.4)	11 (23.9)	17 (37.8)	0.484
ACEI/ARB	127 (69.4)	31 (67.4)	34 (73.9)	32 (69.6)	30 (66.7)	0.876
Beta-blockers	131 (71.6)	30 (65.2)	38 (82.6)	34 (73.9)	29 (64.4)	0.178
ARNI	43 (23.5)	8 (17.4)	14 (30.4)	10 (21.7)	11 (24.4)	0.515
Amiodarone	29 (15.8)	9 (19.6)	7 (15.2)	5 (10.9)	8 (17.8)	0.689
Digoxin	59 (32.2)	11 (23.9)	13 (28.3)	15 (32.6)	20 (44.4)	0.182
Antiplatelet agents	101 (55.2)	29 (63.0)	26 (56.5)	24 (52.2)	22 (48.9)	0.558
Anticoagulants	79 (43.2)	16 (34.8)	18 (39.1)	24 (52.2)	21 (46.7)	0.338
Statins	111 (60.7)	33 (71.1)	29 (63.0)	21 (45.7)	28 (62.2)	0.076
Insulin	15 (8.2)	3 (6.5)	5 (10.9)	4 (8.7)	3 (6.7)	0.858
Oral hypoglycemic agents	39 (21.3)	8 (17.4)	12 (26.1)	7 (15.2)	12 (26.7)	0.418
Trimetazidine	37 (20.2)	7 (15.2)	10 (21.7)	11 (23.9)	9 (20.0)	0.760
Calcium antagonists	32 (17.5)	8 (17.4)	11 (23.9)	7 (15.2)	6 (13.3)	0.568
Laboratory test						
NT-proBNP, pg/mL	4264.0 (2449.0, 8054.0)	3025.5 (1590.6, 5529.8)	3525.0 (2156.8, 5250.3)	5989.1 (2719.8, 9570.3)	5167.3 (3191.5, 9483.0)	**0.001**
ALT, U/L	28.0 (17.0, 43.0)	22.0 (15.0, 34.3)	26.0 (15.0, 35.0)	31.0 (17.5, 45.0)	36.0 (21.5,57.5)	**0.022**
AST, U/L	39.0 (29.0, 59.0)	32.5 (25.0, 49.3)	38.5 (31.0, 58.0)	39.0 (29.8, 52.0)	46.0 (29.5, 69.5)	0.051
BUN, mmol/L	8.4 (7.2, 9.6)	8.3 (7.2, 9.5)	8.8 (7.4, 9.7)	8.8 (7.4, 9.7)	7.7 (6.5, 9.0)	0.135
Creatinine, μmol/L	91.0 (81.0, 98.0)	92.0 (80.5, 99.0)	90.5 (83.0, 100.0)	92.0 (81.8, 98.0)	88.0 (79.5, 96.5)	0.669
eGFR, mL/min·1.73 m^2^	66.9 ± 18.4	65.3 ± 20.2	65.4 ± 18.0	64.7 ± 17.5	72.3 ± 17.4	0.157
Total cholesterol, mmol/L	3.8 ± 1.0	4.1 ± 1.0	3.5 ± 1.0	3.8 ± 0.8	3.8 ± 1.0	**0.032**
LDL-C, mmol/L	2.0 ± 0.7	2.1 ± 0.7	1.7 ± 0.6	2.0 ± 0.5	2.0 ± 0.7	**0.038**
HDL-C, mmol/L	1.1 (0.9, 1.3)	1.2 (1.0, 1.3)	1.0 (0.9, 1.3)	1.0 (0.9, 1.3)	1.1 (0.8, 1.2)	0.072
Triglycerides, mmol/L	1.0 (0.7, 1.3)	1.0 (0.7, 1.5)	1.0 (0.7, 1.3)	0.9 (0.7, 1.2)	1.0 (0.8, 1.5)	0.622
Fasting glucose, mmol/L	5.2(4.7, 6.5)	5.2(4.5, 5.8)	5.4(4.7, 8.3)	5.2(5.0, 5.9)	5.2(4.7, 7.9)	0.684
TyG index	8.4(8.1, 8.7)	8.4(8.1, 9.0)	8.5(8.0, 8.8)	8.4(8.1, 8.6)	8.5(8.1, 8.8)	0.669
HbA1c, %	6.2 (5.8, 7.1)	6.1 (5.8, 6.9)	6.3 (5.7, 7.4)	6.2 (5.7, 6.8)	6.2 (5.9, 7.1)	0.708
Incidence of all-cause death or HF rehospitalization through the 1- year follow-up	71 (38.8)	12 (26.1)	18 (39.1)	17 (37.0)	24 (53.3)	0.066

*Continuous variables are presented as median (interquartile range) or mean (standard deviation), Categorical variables are expressed as number (percentages). MAP, mean arterial pressure; LVEF, left ventricular ejection fraction; NYHA, New York Heart Association; ACEI/ARB, angiotensin-converting enzyme inhibitor/angiotensin receptor blocker; ARNI, angiotensin receptor-neprilysin inhibitor; NT-proBNP, N-terminal pro brain natriuretic peptide; ALT, alanine aminotransferase; AST, aspartate amino transferase; BUN, urea nitrogen; eGFR, estimated glomerular filtration rate; LDL-C, low-density lipoprotein cholesterol; HDL-C, high-density lipoprotein cholesterol; TyG index, Triglyceride-glucose index; HbA1c, glycated hemoglobin A1c. Bold font indicates statistical significance*.

### Correlation of FFAs With Baseline Characteristics

As illustrated in Table 2, the serum levels of FFAs showed significant positive correlations with NT-proBNP, NYHA. A negative correlation between FFAs levels and LVEF was also implicated. Besides, the FFAs levels were correlated with age, eGFR, ALT, and AST but did not show any significant correlation with BMI and MAP. Although the total serum lipid levels did not show any correlation with FFAs, HDL-cholesterol was negatively associated with FFAs instead of any other components of serum lipids. Notably, we saw no significant association between FFAs and TyG index, the reliable surrogate marker of insulin resistance (IR) (17).

**Table 2:** Correlation analyses of free fatty acids with clinical and laboratory parameters.

	**FFAs**	
	***r***	***P*-value**
Age, years	**−0.175**	**0.018**
BMI, Kg/m^2^	0.114	0.126
MAP, mmHg	0.085	0.252
NYHA	**0.207**	**0.005**
NT-pro BNP, pg/mL	**0.287**	**<0.001**
LVEF, %	**−0.193**	**0.009**
ALT, U/L	**0.241**	**0.001**
AST, U/L	**0.203**	**0.006**
eGFR, mL/min·1.73 m^2^	**0.160**	**0.030**
Total cholesterol, mmol/L	−0.050	0.498
LDL-C, mmol/L	0.032	0.670
HDL-C, mmol/L	**−0.173**	**0.019**
Triglycerides, mmol/L	−0.018	0.810
Fasting glucose, mmol/L	0.037	0.615
HbA1c	0.087	0.262
TyG index	0.001	0.989

*BMI, body-mass index; MAP, mean arterial pressure; LVEF, left ventricular ejection fraction; NYHA, New York Heart Association; NT-proBNP, N-terminal pro brain natriuretic peptide; ALT, alanine aminotransferase; AST, aspartate amino transferase; eGFR, estimated glomerular filtration rate; LDL-C, low-density lipoprotein cholesterol; HDL-C, high-density lipoprotein cholesterol; TyG index, Triglyceride-glucose index; HbA1c, glycated hemoglobin A1c. Bold font indicates statistical significance*.

### Association of FFAs With Adverse Events

**Figure 1:**
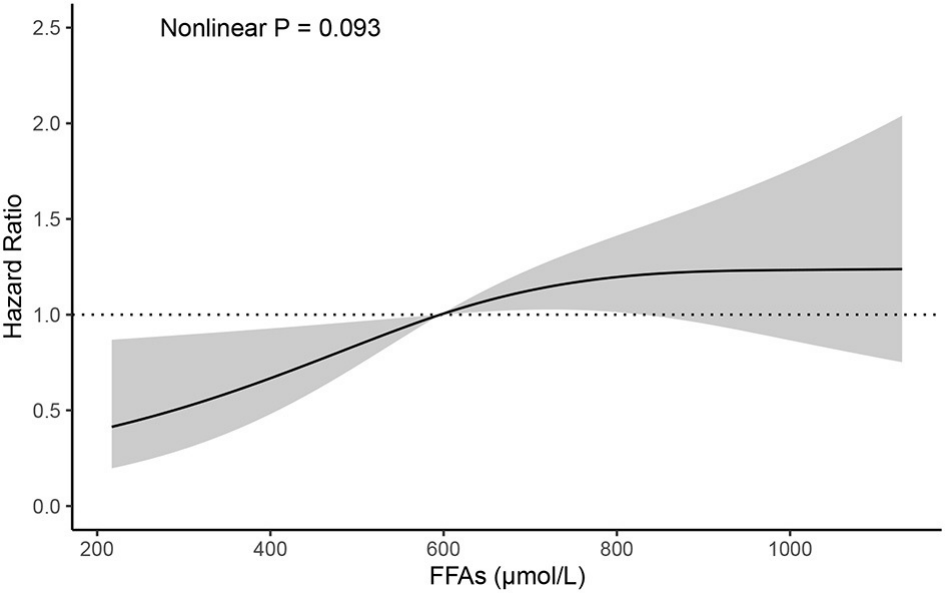
Cubic spline depicting the association of FFAs with the incidence of all-cause mortality or HF rehospitalization, P non-liner = 0.093 (adjusted for age, sex, BMI, CAD, DM, hypertension, LVEF, NT-proBNP). BMI, body-mass index; CAD, coronary artery disease; DM, diabetes mellitus; LVEF, left ventricular ejection fraction; NT-proBNP, N-terminal pro brain natriuretic peptide.

During the 1-year flow-up after discharge, 71 out of a total of 183 AHF patients (38.8%) experienced an adverse event (all-cause death or HF rehospitalization). After adjusting for variables that may influence the prognosis in HF and the incidence of all-cause death or HF rehospitalization analyzed by the univariate analysis in the present study (Supplementary Table 2) (18–21), there was a positive association between FFAs levels and the risk of death or HF rehospitalization (Table 3). In a multivariable analysis adjusting for sex, age, body-mass index, coronary artery disease, diabetes mellitus, hypertension, LVEF, NT-proBNP, each SD (303.07 μmol/L) higher FFAs were associated with 26% higher risk of death or HF rehospitalization (95% confidence interval, 2–55%). Each increasing quartile of FFAs was associated with differentially elevated hazard ratios for death or HF rehospitalization of 1 (reference), 1.71 (95% confidence interval, [0.81, 3.62]), 1.41 (95% confidence interval, [0.64, 3.09]), and 3.18 (95% confidence interval, [1.53, 6.63]), respectively. Assessment of cubic splines also supports a linear relationship between the serum levels of FFAs and the incidence of death or HF rehospitalization (P non-liner = 0.093) (Figure 1).

**Table 3:** Univariate and multivariate Cox regression model for all-cause death or HF rehospitalization according to quartiles/standard deviation of serum free fatty acids.

**FFAs range**	**Free Fatty Acids Quartiles**				**Continuous**
	**Q1 (≤385.60)**	**Q2 (385.60–596.20)**	**Q3 (596.20–797.30)**	**Q4 (>797.30)**	**Per standard deviation (303.07) greater**
Events/N at risk	12/46	18/46	17/46	24/45	71/183
Unadjusted HR (95%CI)	1.00(Ref.)	1.60 (0.77, 3.32)	1.51 (0.72, 3.17)	2.71 (1.36, 5.43)	1.24(1.02, 1.51)
Adjusted Model ^*^ HR (95%CI)	1.00(Ref.)	1.71 (0.81, 3.62)	1.41 (0.64, 3.09)	3.18 (1.53, 6.63)	1.26 (1.02, 1.55)

* *Adjusted for age, sex, BMI, CAD, DM, hypertension, LVEF, NT-proBNP. BMI, body-mass index; CAD, coronary artery disease; DM, diabetes mellitus; LVEF, left ventricular ejection fraction; NT-proBNP, N-terminal pro brain natriuretic peptide*.

## Discussion

Our study shows that serum FFAs concentration is an independent risk factor for all-cause death or HF rehospitalization after discharge of patients with AHF, indicating that FFAs may predict adverse outcomes in AHF patients.

Øie et al. conducted a cross-sectional study, which showed that 183 patients with stable HF had higher FFAs levels than that in healthy control subjects (11). Previous studies have reported that circulating FFAs were associated with various risks factors for HF including coronary heart disease (9, 10), hypertension (22), diabetes mellitus (8), atrial fibrillation (23). This would lead to a higher risk of HF (12) and higher 3-month mortality of HF (13). Accumulating evidence showed that Asian/Chinese population may have a different fatty acid metabolism pattern (24). Our study not only provided direct evidence with the positive correlation between serum FFAs and acute heart failure, but also for the first time showed that FFAs may have good prognostic value in predicting adverse events in Chinese population. Moreover, this prognostic value may last longer than we expected, which may be more than 1 years. All these studies, with our findings together, call for a future study whether diet management, energetic therapy, or metabolic modulation may have therapeutic potential.

FFAs are primarily derived from lipolysis of adipose tissue (25). Some hormones, such as catecholamines and inflammatory cytokines, which can exert strong lipolytic signals on hormone-sensitive lipase, were found markedly elevating in HF patients, contributing to the enhanced concentration of FFAs in circulation (14). Since the rate of fatty acids (FAs) uptake by the heart is mainly dependent upon the concentration of FFAs in the circulation, FAs delivery to cardiomyocytes are also increased along with the growing concentration of FFAs (26). Of note, an increased FAs uptake is not accompanied by a concomitant augmented FA oxidation. Indeed, evidence is mounting that deteriorated cardiac function comes with a decline in FA oxidation rates (27, 28). The imbalance between FA uptake and oxidation results in intracellular storage of lipids, which are in part stored as triglycerides (TAGs), but can also be transported into non-oxidative pathways, leading to the production of toxic lipid species like diacylglycerol and ceramide, which drives the cardiac lipotoxicity (4). The cardiac lipotoxicity, inefficient FA metabolism, together with the negative impact of high FFAs on cardiovascular system including insulin resistance, oxidative stress, inflammation and endothelial dysfunction (29, 30), indicates that elevated FFAs play an important role in HF pathophysiology and might contribute to the development and progression of HF. Notably, Studies in humans and animal models have revealed that heart failure is associated with generalized insulin resistance (31). And evidence showed that excess of circulating FFAs are the major cause of IR by inhibiting insulin signaling (32, 33). Unexpectedly, we found no significant association between FFAs and TyG index, the reliable surrogate of IR, to some extent suggesting that the relationship of FFAs and insulin resistance is more complex in HF patients, at least in the acute phase, which deserves further investigation. Our data suggests a positive correlations of serum levels of FFAs with NYHA and NT-proBNP, in accord with the results of a previous study (34), verifying again the relationship between serum FFAs levels and HF severity.

Given the facts listed above, and the highly significant association between FFAs levels and the adverse clinical outcomes among AHF patients, as presented in our study and a previous study (13), it seems reasonable to suppose that modulation of FFAs utilization by application of medicines might improve the cardiac function and outcomes in HF patients. Perhexiline, reducing FAO through inhibiting carnitine palmitoyltransferase-1 and 2 (CPT-1/2), the transport proteins responsible for mitochondrial FAs uptake from the cytoplasm (35), was found that could improve symptoms, the peak exercise oxygen consumption (VO2 max) and LVEF in patients with chronic HF (36–39). Trimetazidine, acting as a inhibitor of long-chain mitochondrial 3-ketoacyl coenzyme A thiolase enzyme, leading to the reduced myocardial FA oxidation (40), improved left ventricular (LV) function and functional class, reduced rehospitalization rates (41–43) and all-cause mortality (43–45) in HF patients. However, different results also have been reported. Use of acipimox, a nicotinic acid analog, did not result in the same favorable effect on myocardial function among HF patient, which can reduce the availability of circulating FFAs through inhibition of adipose tissue lipolysis. Four-week administration of acipimox in non-diabetic patients with chronic HF did not change cardiac function or exercise capacity (46). What's more, Tuunanen H et al. showed that acute serum FFAs depletion contributed to the deteriorated myocardial efficiency in idiopathic dilated cardiomyopathy patients (47). It's worth noting that these studies did not include AHF patients. AHF patients may have had experienced a more deranged change of myocardial metabolism when compared to stable HF patient, which may respond differently to the limitation of FFAs disposal. Therefore, it is necessary to further explore the mechanism of FFAs in contributing to the development and progression of both stable HF and AHF patients.

### Study Limitations

Our study was an observational study, while its relatively small sample size and single-center in design might introduce selective bias and therefore limit its clinical application. Besides that, the small study sample size precluded us from adequately powered subgroup analysis (presented in Supplementary Material). Moreover, a single measurement of serum FFAs failed to provide information about the longitudinal changes in FFAs levels over time and how the changes affect the clinical outcomes of AHF patients. A caveat of our study was that we were unable to detect the composition of serum FFAs but the total class for all the non-esterized fatty acids. Previous data suggested that FFAs composition may influence myocardial function and associated with total mortality in chronic heart failure population (11) and linked to the incidence of HF in meddle-aged adults (48). However, evidence for an association between the composition of FFAs and AHF is still limited, which warrant further investigation. A more precise evaluation about the specific components of FFAs may give a deeper insight of cardiac energetics and help to seek more specific therapeutic targets. In future, basic researches are in great need to clarify the role of FFAs in the development and progression of HF.

## Conclusion

In conclusion, our data demonstrated an increased risk of adverse clinical outcomes with higher FFAs concentration among AHF patients. FFAs levels, which can be easily measured in clinical setting at relatively lower costs, may have a great prognostic potential for risk stratification in AHF patients.

## Data Availability Statement

The original contributions presented in the study are included in the article/Supplementary Materials, further inquiries can be directed to the corresponding authors.

## Ethics Statement

The studies involving human participants were reviewed and approved by the Institutional Review Board for Human Studies of Second Affiliated Hospital of Zhejiang University School of Medicine (Hangzhou, China). Written informed consent for participation was not required for this study in accordance with the national legislation and the institutional requirements.

## Author Contributions

YY performed data collection, patient follow-up, statistical analysis and manuscript writing. CJ participated in statistical analysis. CZ participated in data collection. SZ and SM participated in data collection and analysis. HM conceived the study and revised the manuscript. MX and JW provided funding and overall supervision. All authors contributed to the article and approved the submitted version.

## Funding

This article was supported by Provincial and Ministry Joint Major Projects of National Health Commission of China (WKJ-ZJ-1703 to MX), the Key Research and Development Project of Department of Science and Technology of Zhejiang Province (2020C03118 to MX), National Natural Science Foundation of China (82070251 and 81870203 to MX, 82070252 to HM) and grants from and Zhejiang Provincial Natural Science Foundation (LR21H020001 to HM).

## Conflict of Interest

The authors declare that the research was conducted in the absence of any commercial or financial relationships that could be construed as a potential conflict of interest.

## Publisher's Note

All claims expressed in this article are solely those of the authors and do not necessarily represent those of their affiliated organizations, or those of the publisher, the editors and the reviewers. Any product that may be evaluated in this article, or claim that may be made by its manufacturer, is not guaranteed or endorsed by the publisher.
