# Supplementary material for: Serum Free Fatty Acids Independently Predict Adverse Outcomes in Acute Heart Failure Patients
Source: Front Cardiovasc Med. 2021 Dec 22;8:761537. doi: 10.3389/fcvm.2021.761537 (PMC8727366; doi:10.3389/fcvm.2021.761537)
Supplement: Supplementary file 1 [file Data_Sheet_1.docx]

**Figure S1** Flow chart

**Exclusion**

1.younger than 18 years old（n=0）

2.severe renal failure (eGFR 30ml/min/1.73m^2), renal replacement therapy, severe liver dysfunction (serum aminotransferase concentration more than 10 times above upper limit of normal range) (n=36)

3.serious infection, severe pulmonary diseases（n=11）

4.systemic autoimmune disorder (n=4)

5.malignancy (n=11)

6.pregnancy (n=0)

7.FFA missing（n=9）

8.severe valvular disease（n=33）

9.patients who died in hospital (n=1)

10. patients lost to 1-year follow-up (n=16)

**Inclusion**

patients admitted for AHF in the second affiliated hospital Zhejiang university school of medicine Department of Cardiology between January 2019 and December 2019 were enrolled.

The diagnosis of AHF was established based on integrated clinical judgment according to the ESC Guidelines and required elevated N-terminal pro-B-type natriuretic peptide (NT-proBNP) concentrations of at least 1000ng/L and only those whose duration from onset of AHF or symptom exacerbation was≤1 month were recruited.

medical records review

final cohort (n=183)

included patients（n=304）

**Table S1 Baseline Characteristics Stratified by Study Events**

|  | **All patients**  **(n=183)** | **Event-free**  **(n = 112)** | **Event**  **(N = 71)** | **P-value** |
| --- | --- | --- | --- | --- |
| **Age, years** | 73 (63, 79) | 72 (63, 79) | 74 (64, 80) | 0.473 |
| **Male sex, *n* (%)** | 117 (63.9%) | 70 (62.5%) | 47 (66.2%) | 0.612 |
| **BMI, kg/m²** | 23.8  (20.9, 26.0) | 23.9  (21.8, 26.0) | 23.8  (21.0, 26.1) | 0.934 |
| **MAP, mmHg** | 91 (82, 100) | 92.3  (84.3, 104.2) | 88.7  (80.0, 96.7) | **0.033** |
| **LVEF, %** | 35 (28, 48) | 36.1  (28.1, 54.1) | 34.4  (29.0, 43.3) | 0.474 |
| **NYHA functional class** |  |  |  | 0.736 |
| **II, *n* (%)** | 33 (18.0%) | 20 (17.9%) | 13 (18.3%) |  |
| **III, *n* (%)** | 81 (44.3%) | 52 (46.4%) | 29 (40.8%) |  |
| **IV, *n* (%)** | 69 (37.7%) | 40(35.7%) | 29 (40.8%) |  |
| ***De novo* HF, n (%)** | 47 (25.7%) | 34 (30.4%) | 13 (18.3%) | 0.069 |
| **Co-morbidities, *n* (%)** |  |  |  |  |
| **Coronary artery disease** | 71 (38.8%) | 38 (33.9%) | 33 (46.5%) | 0.090 |
| **Diabetes mellitus** | 51 (27.9%) | 29 (25.9%) | 22 (31.0%) | 0.454 |
| **Hypertension** | 98 (53.6%) | 58 (51.8%) | 40 (56.3%) | 0.547 |
| **Atrial fibrillation/ atrial flutter** | 87 (47.5%) | 55 (49.1%) | 32 (45.1%) | 0.594 |
| **Smoking** | 67 (36.6%) | 39 (34.8%) | 28 (39.4%) | 0.528 |
| **Treatment, *n* (%)** |  |  |  |  |
| **Intravenous Diuretics** | 128 (69.9%) | 76 (67.9%) | 52(73.2%) | 0.439 |
| **Intravenous vasodilator** | 15 (8.2%) | 11 (9.8%) | 4 (5.6%) | 0.314 |
| **Intravenous vasopressor** | 6 (3.3%) | 3 (2.7%) | 3 (4.2%) | 0.567 |
| **Intravenous inotropic agent** | 54 (29.5%) | 33 (29.5%) | 21 (39.6%) | 0.987 |
| **ACEI/ARB** | 127 (69.4%) | 76 (67.9%) | 51 (71.8%) | 0.570 |
| **Beta-blockers** | 131 (71.6%) | 81(72.3%) | 50 (70.4%) | 0.781 |
| **ARNI** | 43 (23.5%) | 30 (26.8%) | 13 (18.3%) | 0.188 |
| **Amiodarone** | 29 (15.8%) | 16 (14.3%) | 13 (18.3%) | 0.468 |
| **Digoxin** | 59 (32.2%) | 34 (30.4%) | 25(35.2%) | 0.494 |
| **Antiplatelet agents** | 101 (55.2%) | 60 (53.6%) | 41 (57.7%) | 0.580 |
| **Anticoagulants** | 79 (43.2%) | 51(45.5%) | 28 (39.4%) | 0.417 |
| **Statins** | 111 (60.7%) | 69 (61.6%) | 42 (59.2%) | 0.741 |
| **Insulin** | 15 (8.2%) | 8 (7.1%) | 7 (9.9%) | 0.514 |
| **Oral hypoglycemic agents** | 39 (21.3%) | 22 (19.6%) | 17 (23.9%) | 0.489 |
| **Trimetazidine** | 37 (20.2%) | 21(18.8%) | 16 (22.5%) | 0.534 |
| **CCB** | 32 (17.5%) | 22 (19.6%) | 10 (14.1%) | 0.335 |
| **Laboratory test results** |  |  |  |  |
| **FFAs, mmol/L** | 596.2  (385.7,796.8) | 547.3 (323.5,746.5) | 665.4  (471.6,850.8) | **0.022** |
| **NT-proBNP, pg/mL** | 4264.0  (2,453.5, 8,037.5) | 3736.5 (2212.3, 6864.3) | 4986.0 (2647.0, 8534.8) | 0.112 |
| **ALT, U/L** | 28.0  (17.5, 42.5) | 28.0  (17.3, 43.8) | 30.0  (17.0, 41.0) | 0.992 |
| **AST, U/L** | 39.0 (29.5, 58.5) | 40.0  (30.3, 61.8) | 37.0 (27.0, 56.0) | 0.204 |
| **BUN, mmol/L** | 8.4 (7.2, 9.5) | 8.4 (7.1, 9.5) | 8.3 (7.2, 9.7) | 0.780 |
| **Creatinine, umol/L** | 91.0 (81.0, 98.0) | 89.5 (80.0, 98.0) | 91.0 (84.0, 98.0) | 0.490 |
| **eGFR, mL/min·1.73m^2^** | 66.5 (54.2, 78.9) | 67.8 (54.2, 81.9) | 64.5 (53.8, 77.9) | 0.365 |
| **Total cholesterol, mmol/L** | 3.8 (3.1, 4.3) | 3.8 (3.2, 4.3) | 3.9(3.0, 4.2) | 0.586 |
| **LDL-C, mmol/L** | 1.9 (1.4, 2.4) | 1.9 (1.4, 2.3) | 1.8 (1.4, 2.4) | 0.788 |
| **HDL-C, mmol/L** | 1.1 (0.9, 1.3) | 1.1 (0.9, 1.3) | 1.1 (0.9, 1.2) | 0.377 |
| **Triglycerides, mmol/L** | 1.0 (0.7, 1.3) | 1.0 (0.8, 1.4) | 0.9 (0.7, 1.3) | **0.049** |
| **Fasting glucose, mmol/L** | 5.2 (4.7, 6.5) | 5.3 (4.7, 6.8) | 5.1 (4.6, 6.4) | 0.503 |
| **TyG index** | 8.4 (8.1, 8.7) | 8.4 (8.1, 9.0) | 8.4(8.0, 8.7) | 0.100 |
| **HbA1c, %** | 6.2 (5.8, 7.0) | 6.1 (5.7, 7.0) | 6.2 (5.9, 7.1) | 0.274 |

Continuous variables are presented as median (interquartile range) or mean (standard deviation), Categorical variables are expressed as number (percentages).

Abbreviations: MAP, mean arterial pressure; LVEF, left ventricular ejection fraction; NYHA, New York Heart Association; ACEI/ARB, angiotensin-converting enzyme inhibitor/angiotensin receptor blocker; ARNI, angiotensin receptor-neprilysin inhibitor; FFA, free fatty acid; NT-proBNP, N-terminal pro brain natriuretic peptide; ALT, alanine aminotransferase; AST, aspartate amino transferase; BUN, urea nitrogen; eGFR, estimated glomerular filtration rate; LDL-C, low-density lipoprotein cholesterol; HDL-C, high-density lipoprotein cholesterol; TyG index, Triglyceride-glucose index; HbA1c, glycated hemoglobin A1c.

**Table S2** Univariate Cox Proportional Hazard Model

|  | **P-value** |  | **P-value** |
| --- | --- | --- | --- |
| **Age** | 0.360 | **Lab data** |  |
| **sex** | 0.488 | **NT-proBNP** | 0.724 |
| **BMI (m²)** | 0.932 | **ALT, U/L** | 0.791 |
| **MAP (mmHg)** | **0.058** | **AST, U/L** | 0.354 |
| **EF (%)** | 0.345 | **BUN, mmol/L** | 0.933 |
| **NYHA** |  | **Creatinine, mol/L** | 0.744 |
| **class 2**, ***n* (%)** | Ref. | **eGFR, mL/min·1.73m^2^** | 0.508 |
| **class 3**, ***n* (%)** | 0.586 | **Total cholesterol, mmol/L** | 0.439 |
| **class 4**, ***n* (%)** | 0.843 | **LDL cholesterol, mmol/L** | 0.739 |
| **Risk factors or Co-morbidities, *n* (%)** |  | **HDL cholesterol, mmol/L** | 0.576 |
| **Coronary artery disease** | **0.069** | **Triglycerides, mmol/L** | **0.069** |
| **Diabetes mellitus** | 0.470 | **Fasting glucose, mmol/L** | 0.242 |
| **Hypertension** | 0.777 | **HbA1c, %** | 0.404 |
| **Atrial fibrillation/ atrial flutter** | 0.598 |  |  |
| **Smoking** | 0.433 |  |  |
| **Medications during hospitalization, *n* (%)** |  |  |  |
| **IV Diuretics** | 0.428 |  |  |
| **IV vasodilator** | 0.337 |  |  |
| **IV vasopressor** | 0.432 |  |  |
| **IV inotropic agent** | 0.778 |  |  |
| **ACEI/ARB** | 0.617 |  |  |
| **Beta-blockers** | 0.718 |  |  |
| **ARNI** | 0.234 |  |  |
| **Amiodarone** | 0.587 |  |  |
| **Digoxin** | 0.498 |  |  |
| **antiplatelet therapy** | 0.528 |  |  |
| **Anticoagulants** | 0.414 |  |  |
| **Statins** | 0.845 |  |  |
| **Insulin** | 0.569 |  |  |
| **Oral hypoglycemic agent** | 0.592 |  |  |
| **Trimetazidine** | 0.486 |  |  |
| **CCB** | 0.409 |  |  |

Abbreviations: MAP, mean arterial pressure; LVEF, left ventricular ejection fraction; NYHA, New York Heart Association; ACEI/ARB, angiotensin-converting enzyme inhibitor/angiotensin receptor blocker; ARNI, angiotensin receptor-neprilysin inhibitor; FFA, free fatty acid; NT-proBNP, N-terminal pro brain natriuretic peptide; ALT, alanine aminotransferase; AST, aspartate amino transferase; BUN, urea nitrogen; eGFR, estimated glomerular filtration rate; LDL-C, low-density lipoprotein cholesterol; HDL-C, high-density lipoprotein cholesterol; IV, intravenous; HbA1c, glycated hemoglobin A1c.

**Table S3 Baseline characteristics according to de novo HF/decompensated CHF**

|  | **All patients**  **n=183** | **De novo HF**  **n=47** | **Decompensated CHF n=136** | **P-value** |
| --- | --- | --- | --- | --- |
| **Age, years** | 73 (63,79) | 70(22,26) | 74(65,81) | 0.055 |
| **Male sex, *n* (%)** | 117 (63.9) | 33 (70.2) | 84 (61.8) | 0.379 |
| **Body-mass index, kg/m²** | 23.8 (20.8,26.0) | 24.2(21.6,26.2) | 23.7(20.8,25.6) | 0.179 |
| **MAP, mmHg** | 90.7 (82.0,100.7) | 93.3(85.0,105.0) | 89.8(81.2,98.7) | 0.054 |
| **LVEF, %** | 35.3 (28.2,48.5) | 35.9(28.2,51.7) | 35.2(28.1,48.0) | 0.991 |
| **NYHA functional class** |  |  |  | 0.958 |
| **II, *n* (%)** | 33 (18.0) | 9 (19.1) | 24 (17.6) |  |
| **III, *n* (%)** | 81 (44.3) | 21 (44.7) | 60 (44.1) |  |
| **IV, *n* (%)** | 69 (37.7) | 17 (36.2) | 52 (38.2) |  |
| **Co-morbidities, *n* (%)** |  |  |  |  |
| **Coronary artery disease** | 71 (38.8) | 17 (36.2) | 54 (39.7) | 0.730 |
| **Diabetes mellitus** | 51 (27.9) | 15 (31.9) | 36 (26.5) | 0.572 |
| **Hypertension** | 98 (53.6) | 25 (53.2) | 73 (53.7) | 1.000 |
| **Atrial fibrillation/ atrial flutter** | 87 (47.5) | 14 (29.8) | 73 (53.7) | 0.006 |
| **Smoking** | 67 (36.6) | 16 (34.0) | 51 (37.5) | 0.728 |
| **Medications during hospitalization, *n* (%)** |  |  |  |  |
| **Intravenous Diuretics** | 128 (69.9) | 33 (70.2) | 95 (69.9) | 1.000 |
| **Intravenous vasodilator** | 15 (8.2) | 6 (12.8) | 9 (6.6) | 0.218 |
| **Intravenous vasopressor** | 6 (3.3) | 2 (4.3) | 4 (2.9) | 0.648 |
| **Intravenous inotropic agent** | 54 (29.5) | 13 (27.7) | 41 (30.1) | 0.854 |
| **ACEI/ARB** | 127 (69.4) | 35 (74.5) | 92 (67.6) | 0.464 |
| **Beta-blockers** | 131 (71.6) | 36 (76.6) | 95 (69.9) | 0.455 |
| **ARNI** | 43 (23.5) | 10 (21.3) | 33 (24.3) | 0.842 |
| **Amiodarone** | 29 (15.8) | 4 (8.5) | 25 (18.4) | 0.163 |
| **Digoxin** | 59 (32.2) | 18 (38.3) | 41 (30.1) | 0.366 |
| **Antiplatelet agents** | 101 (55.2) | 29 (61.7) | 72 (52.9) | 0.313 |
| **Anticoagulants** | 79 (43.2) | 15 (31.9) | 64 (47.1) | 0.088 |
| **Statins** | 111 (60.7) | 33 (70.2) | 78 (57.4) | 0.165 |
| **Insulin** | 15 (8.2) | 5 (10.6) | 10 (7.4) | 0.539 |
| **Oral hypoglycemic agents** | 39 (21.3) | 13 (27.7) | 26 (19.1) | 0.222 |
| **Trimetazidine** | 37 (20.2) | 7 (14.9) | 30 (22.1) | 0.400 |
| **Calcium antagonists** | 32 (17.5) | 6 (12.8) | 26 (19.1) | 0.380 |
| **Laboratory test** |  |  |  |  |
| **Free fatty acids, mmol/L** | 621.8±303.1 | 623.1±304.0 | 621.3±303.8 | 0.971 |
| **NT-proBNP, pg/mL** | 4264.0 (2449.0,8054.0) | 4098.0 (2449.0,5611.0) | 4303.8 (2418.3,8106.3) | 0.541 |
| **ALT, U/L** | 28.0 (17.0,43.0) | 30.0 (18.0,47.0) | 27.5 (16.0,41.0) | 0.469 |
| **AST, U/L** | 39.0 (29.0,59.0) | 39.0 (31.0,65.0) | 39.0 (28.3,55.8) | 0.575 |
| **BUN, mmol/L** | 8.4 (7.2,9.6) | 7.7 (6.7,9.1) | 8.6 (7.3,9.6) | 0.017 |
| **Creatinine, umol/L** | 91.0 (81.0,98.0) | 89.0 (82.0,97.0) | 91.0(80.3,99.0) | 0.624 |
| **eGFR, mL/min·1.73m^2^** | 66.9±18.4 | 71.4±18.2 | 65.3±18.3 | 0.053 |
| **Total cholesterol, mmol/L** | 3.8±1.0 | 4.0±1.1 | 3.7±0.9 | 0.131 |
| **LDL-C, mmol/L** | 2.0±0.7 | 2.08±0.8 | 1.9±0.6 | 0.116 |
| **HDL-C, mmol/L** | 1.1 (0.9,1.3) | 1.1 (0.9,1.3) | 1.1(0.9,1.3) | 0.971 |
| **Triglycerides, mmol/L** | 1.0 (0.7,1.3) | 1.0 (0.8,1.3) | 1.0 (0.7,1.3) | 0.619 |
| **Fasting glucose, mmol/L** | 5.2 (4.7,6.5) | 5.4 (4.7,8.1) | 5.2 (4.7,6.4) | 0.487 |
| **TyG index** | 8.4 (8.1,8.7) | 8.5 (8.1,8.7) | 8.4 (8.1,8.7) | 0.335 |
| **HbA1c, %** | 6.2 (5.8,7.1) | 6.1 (5.8,7.2) | 6.2 (5.8,7.0) | 0.874 |
| **Incidence of all-cause death or HF rehospitalization through the 1- year follow-up** | 71 (38.8) | 13 (27.7) | 58 (42.6) | 0.083 |

Abbreviations: MAP, mean arterial pressure; LVEF, left ventricular ejection fraction; NYHA, New York Heart Association; ACEI/ARB, angiotensin-converting enzyme inhibitor/angiotensin receptor blocker; ARNI, angiotensin receptor-neprilysin inhibitor; FFA, free fatty acid; NT-proBNP, N-terminal pro brain natriuretic peptide; ALT, alanine aminotransferase; AST, aspartate amino transferase; BUN, urea nitrogen; eGFR, estimated glomerular filtration rate; LDL-C, low-density lipoprotein cholesterol; HDL-C, high-density lipoprotein cholesterol; IV, intravenous; HbA1c, glycated hemoglobin A1c.

**Table S4 De novo HF**

|  | **Continuous** |
| --- | --- |
| **FFAs range** | **Per standard deviation**  **(303.98) greater** |
| **Events/N**  **at risk** | 13/47 |
| **Unadjusted HR (95%CI)** | 1.43(0.86,2.40) |
| **Adjusted Model * HR (95%CI)** | 1.40  (0.70,2.81) |

*adjusted for age, sex, BMI, CAD, DM, hypertension，NT-proBNP, LVEF

Abbreviations: BMI, body-mass index; CAD, coronary artery disease; DM, diabetes mellitus; NT-proBNP, N-terminal pro brain natriuretic peptide; LVEF, left ventricular ejection fraction

**Table S5 Decompensated CHF**

|  | **Free Fatty Acids Quartiles** | | | | **Continuous** |
| --- | --- | --- | --- | --- | --- |
| **FFAs range** | **Q1**  **(≤387.87)** | **Q2**  **(387.87-606.25)** | **Q3**  **(606.25-797.03)** | **Q4**  **(>797.03)** | **Per standard deviation**  **(303.87) greater** |
| **Events/N**  **at risk** | 11/34 | 14/34 | 14/34 | 19/34 | 58/136 |
| **Unadjusted HR (95%CI)** | 1.00(Ref.) | 1.36  (0.61,2.99) | 1.39  (0.63,3.06) | 2.43  (1.07,4.72) | 1.21(0.98,1.50) |
| **Adjusted Model * HR (95%CI)** | 1.00(Ref.) | 1.37  (0.61,3.06) | 1.36  (0.60,3.09) | 2.54  (1.48,5.64) | 1.26  (1.00,1.60) |

*adjusted for age, sex, BMI, CAD, DM, hypertension，NT-proBNP, LVEF

Abbreviations: BMI, body-mass index; CAD, coronary artery disease; DM, diabetes mellitus; NT-proBNP, N-terminal pro brain natriuretic peptide; LVEF, left ventricular ejection fraction

**Table S6 All-cause death**

|  | **Continuous** |
| --- | --- |
| **FFAs range** | **Per standard deviation**  **(303.07) greater** |
| **Events/N**  **at risk** | 13/183 |
| **Unadjusted HR (95%CI)** | 1.31(0.50,3.43) |
| **Adjusted Model * HR (95%CI)** | 2.13  (0.56,8.04) |

***adjusted for age, sex, BMI, CAD, DM, hypertension，NT-proBNP, LVEF**

**Abbreviations: BMI, body-mass index; CAD, coronary artery disease; DM, diabetes mellitus; NT-proBNP, N-terminal pro brain natriuretic peptide; LVEF, left ventricular ejection fraction**

**Table S7 HF rehospitalization**

|  | **Free Fatty Acids Quartiles** | | | | **Continuous** |
| --- | --- | --- | --- | --- | --- |
| **FFAs range** | **Q1**  **(≤385.60)** | **Q2**  **(385.60-596.20)** | **Q3**  **(596.20-797.30)** | **Q4**  **(>797.30)** | **Per standard deviation**  **(303.07) greater** |
| **Events/N**  **at risk** | 10/46 | 16/46 | 12/46 | 23/45 | 61/183 |
| **Unadjusted HR (95%CI)** | 1.00(Ref.) | 0.54  (0.24,1.23) | 0.70  (0.30,1.66) | 1.49  (0.70,3.16) | 1.49  (1.07,2.06) |
| **Adjusted Model * HR (95%CI)** | 1.00(Ref.) | 0.49  (0.19,1.29) | 0.76  (0.30,1.93) | 1.77  (0.67,4.65) | 1.67  (1.12,2.51) |

*adjusted for age, sex, BMI, CAD, DM, hypertension，NT-proBNP, LVEF

Abbreviations: BMI, body-mass index; CAD, coronary artery disease; DM, diabetes mellitus; NT-proBNP, N-terminal pro brain natriuretic peptide; LVEF, left ventricular ejection fraction

According to the time course of HF, the study population were subgrouped to the new-onset HF, also called de novo HF (n=47) and decompensated chronic HF (n=136). Since the new-onset group were relatively small, FFAs could be only modeled as a continuous variable (per SD of FFAs). As shown in the new table, baseline characteristics were comparable between patients with de novo HF and those with decompensated chronic HF. In our study, only 13 patients died during the 1-year follow-up and the low rate prevented any meaningful statistical estimates of significance. However, there was a positive association between FFAs levels and risk of HF rehospitalization. Multivariable adjusted hazard ratios (95% confidence intervals) for incident of HF rehospitalization were 1.00 (ref.), 1.37 (0.61 to 3.06), 1.36 (0.60 to 3.09), and 2.54 (1.48 to 5.64) from the lowest to highest quartiles of FFAs, respectively.

Figure S2 ROC curve


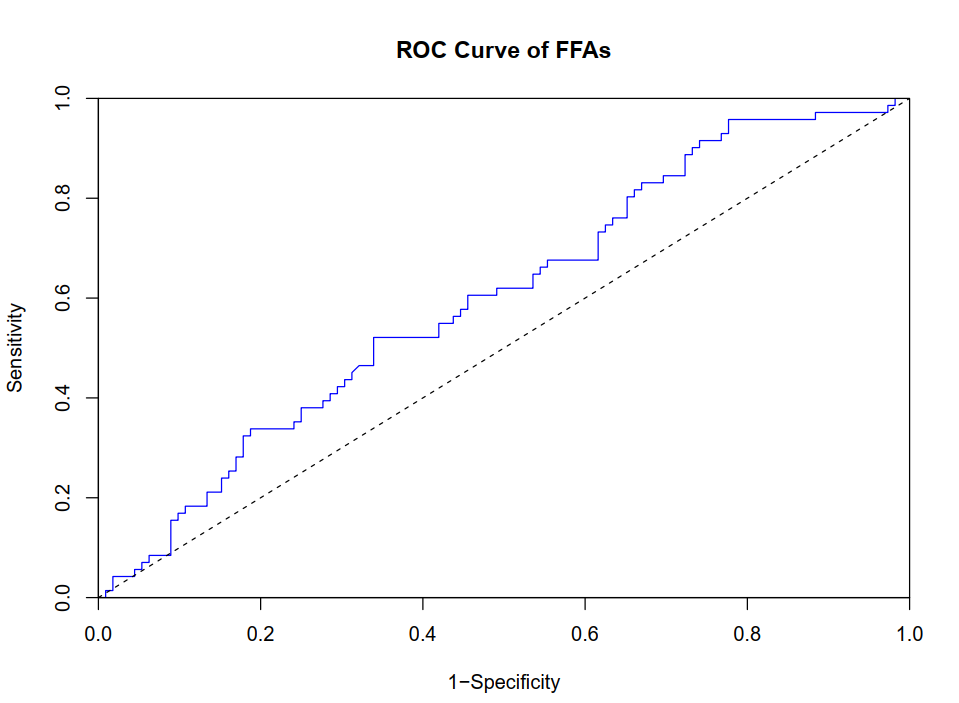


|  | AUC | P-value |
| --- | --- | --- |
| FFAs | 0.601 (0.518,0.684) | 0.022 |

Receiver operating characteristics (ROC) with AUC (area under the curve) for all-cause mortality/HF rehospitalization was 0.601.
